# Epidemiological profiles of patients with chronic migraine and chronic tension-type headache

**DOI:** 10.1186/1129-2377-14-40

**Published:** 2013-05-07

**Authors:** Sara H Schramm, Mark Obermann, Zaza Katsarava, Hans-Christoph Diener, Susanne Moebus, Min-Suk Yoon

**Affiliations:** 1 Institute for Medical Informatics, Biometry and Epidemiology, University Hospital of University Duisburg-Essen, Hufelandstr. 55, 45122 Essen, Germany; 2 Department of Neurology, University Hospital of University Duisburg-Essen, Hufelandstr. 55, Essen, 45122, Germany; 3 Department of Neurology, Evangelisches Krankenhaus Unna, Holbeinstraße 10, Unna, 59423, Germany; 4 Department of Neurology, St. Joseph Hospital, Ruhr-University of Bochum, Gudrunstr. 56, Bochum, 44791, Germany

**Keywords:** Chronic migraine, Chronic tension-type headache, Epidemiology, Risk factors

## Abstract

**Background:**

We evaluated risk factors associated with chronic headache (CH) such as age, gender, smoking, frequent drinking of alcoholic beverages (drinking), obesity, education and frequent intake of acute pain drugs to test their usefulness in clinical differentiation between chronic migraine (CM) and chronic tension-type headache (CTTH).

**Methods:**

We used baseline data from the population-based German Headache Consortium Study including 9,944 participants aged 18–65 years, screened 2003–2005, using validated questionnaires. CM and CTTH were defined according to IHS criteria. Multinominal logistic regression analyses were used to investigate the association of CM or CTTH with risk factors by estimating odds ratios (OR) and 95% confidence intervals (95%CI).

**Results:**

The prevalence of CH was 2.6% (N = 255, mean age 46 ± 14.1 years, 65.1% women), CM 1.1% (N = 108, 45 ± 12.9 years, 73.1%), CTTH 0.5% (N = 50, 49 ± 13.9 years, 48.0%). Participants with CM compared to CTTH were more likely to be female (OR: 2.34, 95%CI: 1.00-5.49) and less likely to drink alcohol (0.31, 0.09-1.04). By trend they seemed more likely to smoke (1.81, 0.76-4.34), to be obese (1.85, 0.54-6.27), to report frequent intake of acute pain drugs (1.68, 0.73-3.88) and less likely to be low educated (0.72, 0.27-1.97).

**Conclusions:**

We concluded that the careful assessment of different risk factors might aid in the clinical differentiation between CM and CTTH.

## Background

Migraine and tension-type headache (TTH) are the most common primary headache disorders affecting up to 80% of the general population [1]. Phenotypically they are different. Migraine is characterized by unilateral severe pulsating headache accompanied by typical autonomic symptoms such as nausea, vomiting, photo- and phonophobia. In contrast, TTH is characterized by mild to moderate headache intensity and of dull, tightening character without autonomic symptoms. Diagnostic criteria are simple and clinically useful in cases with episodic headache but often fail to adequately classify patients with chronic headache that suffer from daily or nearly daily headaches without clearly distinguishable or overlapping clinical features into migraine or tension-type headache. When both conditions coexist in the same patient this problem becomes even more evident. In population based studies in which diagnoses are based on self-reported headache features this unclear distinction might lead to skewered data and result in misinterpretation.

The prevalence of headache subtypes of our study population based on the German Headache Consortium (GHC) was published previously [2]. Due to its large sample size the population of the GHC provides a good opportunity to investigate respondents with pure CM and compares them with pure CTTH in order to investigate differences in possible risk factors such as age, gender, smoking, daily or almost daily drinking of alcoholic beverages (drinking), obesity, education and frequent intake of acute pain drugs that might favour the development of one or the other.

## Methods

### Study design and study population

The study of the GHC was described in detail elsewhere [2]. Briefly, the GHC study is a large population-based cohort study supported by the German Federal Ministry of Education and Research. The study was approved by the ethics committee of the University of Duisburg-Essen, Germany. Informed written consent was obtained from all subjects. 18,000 subjects between the age of 18–65 years with German citizenship were randomly selected via postal mail from statutory lists of residence drawn from three cities in Germany: Essen (585,481 residents, large town), Muenster (272,890 residents, medium-sized town), Sigmaringen (16,501 residents, small town and rural area). We obtained information per mailed questionnaire or telephone interview from 9,944 participants. The questionnaire is based on the ICHD-2 classification criteria of the International Headache Society [3]. A detailed description and validation of the headache-screening questionnaire was published previously [4,5]. In summary, the questionnaire included questions on personal data, inquiry on socio-economic status based primarily on education to avoid direct questions about income, and medical inquiry to diagnose migraine and TTH according to the ICHD-2 classification criteria as well as questions to ascertain the number of days associated with headache and use of acute or preventive medication.

### Analysis

Results are based on the baseline cross sectional analyses of the GHC of 9,944 participants. Primary outcomes were chronic migraine (CM), episodic migraine (EM), chronic tension-type headache (CTTH), episodic tension-type headache (ETTH) and no headache (NH). Migraine was diagnosed if respondents reported headache in the previous year and met ICHD-2 criteria for definite or probable migraine and had either ≥15 headache-days/month (CM) or <15 headache-days/month (EM). TTH was diagnosed if respondents reported headache in the previous year and met ICHD-2 criteria for definite or probable TTH and had either ≥15 headache-days/month (CTTH) or <15 headache-days/month (ETTH).

We investigated the following risk factors: age, gender, smoking, drinking, obesity, education and frequent intake of acute pain drugs. Mean age with standard deviation and gender distribution was calculated for each subgroup. Number of cases and percentages were provided of smoking (yes vs. no), drinking (daily vs. not daily drinking), obesity (Body Mass Index (BMI) >30 kg/m^2^ vs. BMI ≤30 kg/m^2^), education (low vs. high) and frequent intake of acute pain drugs (yes vs. no). Smoking (yes) was defined as current smoking and smoking (no) was defined as never or past smoking. Drinking (yes) was defined as daily or almost daily drinking of alcoholic beverages and drinking (no) was defined as no or casual drinking of alcoholic beverages. High education was defined as completion of secondary (high) school graduation with qualification for University or University diploma and low as any other response. Frequent intake of acute pain drugs was defined as intake of acute pain drugs (including any pain and migraine drugs) on ≥15 days/month.

Descriptive statistics were utilized to characterize the respondent population by headache subtype. Crude odds ratios (OR) and 95% confidence intervals (95%CI) were calculated for each risk factor by headache subtype vs. no headache. Multivariable logistic regression analyses were used to test the association of age, gender, smoking, drinking, obesity and education within headache subtypes or NH by estimating OR and 95%CI (model 1). Frequent intake of acute pain drugs was added to model 1 (model 2). All statistics were completed in SAS, version 9.2 (Statistical Analysis Systems Corp., Cary, NC, USA).

## Results

**Figure 1:**
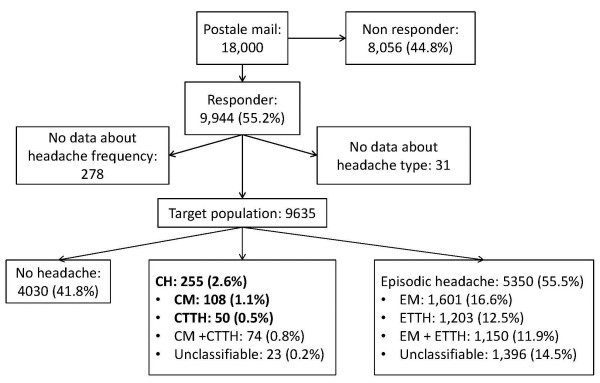
**Flow chart of the study population. **Legend: CH = chronic headache, CM/EM = chronic/episodic migraine, CTTH/ETTH = chronic/episodic tension-type headache, CM+CTTH / EM+ETTH = chronic / episodic migraine with concomitant tension-type headache.

Of 9,944 respondents at baseline 2,952 participants were excluded: 278 of them did not report headache frequency, 31 did not report the headache type, 1,224 had concomitant migraine and TTH and 1,419 had unclassifiable headaches. In 9,635 cases, data about headache subtype were complete. Figure 1 shows the flow chart of our study population and Table 1 shows the characteristics of our study population. We identified 255 participants reporting headache on at least 15 days/month (CH) resulting in a prevalence of 2.6% (95%CI: 2.3-3, mean age 46 ± 14.1 years, 65.1% women). The prevalence of CM was 1.1% (95%CI: 0.9-1.3, N = 108, mean age 45 ± 12.9 years, 73.1% women), of CTTH it was 0.5% (95%CI: 0.4-0.7, N = 50, mean age 49 ± 13.9 years, 48.0% women). In 74 participants we diagnosed chronic migraine with concomitant chronic TTH (CM + CTTH) and in 23 respondents the phenotype of chronic headache was unclassifiable. Overall prevalence of episodic headache was 55.5% (95%CI: 54.5-56.5, N = 5350, mean age 40 ± 12.4 years, 61.6% woman). The prevalence of EM was 16.6% (95%CI: 15.9-17.4, N = 1601, mean age 40 ± 11.8 years, 70.9% women), and of ETTH it was 12.5% (95%CI: 11.8-13.1, N = 1203, mean age 41 ± 12.6 years, 58.0% women). Episodic migraine with concomitant TTH (EM + ETTH) was diagnosed in 1150 participants, unclassifiable episodic headache in 1396 respondents. No headache was reported by 41.8% (95%CI: 40.8-42.8, N = 4030, mean age 47 ± 12.8 years, 40.2% women).

**Table 1:** Characteristics of study population

	**No headache**	**EM**	**CM**	**ETTH**	**CTTH**
	**N = 4030**	**N = 1601**	**N = 108**	**N = 1203**	**N = 50**
**age, years**					
**mean, (SD)**	46.7 (12.8)	40.4 (11.8)	45.3 (12.9)	41.1 (12.6)	49.4 (13.9)
**min, max**	18, 66	5, 66	18, 66	18, 66	19, 65
**missing, N**	2	1	0	1	0
**gender, N (%)**					
**men**	2408 (59.8)	466 (29.1)	29 (26.9)	505 (42.0)	26 (52.0)
**women**	1622 (40.2)	1135 (79.9)	79 (73.1)	698 (58.0)	24 (48.0)
**missing**	0	0	0	0	0
**drinking**^a^**, N (%)**					
**no**	3086 (76.6)	1467 (91.6)	97 (89.8)	1069 (89.0)	38 (76.0)
**yes**	555 (13.8)	106 (6.6)	8 (7.4)	115 (9.5)	11 (22.0)
**missing**	389 (9.6)	28 (1.8)	3 (2.8)	19 (1.5)	1 (2.0)
**smoking**^b^**, N (%)**					
**no**	2581 (64.0)	1071 (66.9)	62 (57.4)	839 (69.7)	32 (64.0)
**yes**	1075 (26.7)	513 (32.0)	44 (40.7)	350 (29.1)	17 (34.0)
**missing**	374 (9.3)	17 (1.1)	2 (1.9)	14 (1.2)	1 (2.0)
**education**^c^**, N (%)**					
high*	1005 (24.9)	548 (34.2)	20 (18.5)	470 (39.1)	12 (24.0)
**low**	2587 (64.1)	1018 (63.6)	86 (79.6)	715 (59.4)	38 (76.0)
**missing**	438 (12.1)	35 (2.2)	2 (1.9)	18 (1.5)	0 (0)
**BMI, N (%)**					
**< 30**	3090 (76.7)	1367 (85.4)	88 (81.5)	1044 (86.8)	43 (86.0)
**≥ 30**	479 (11.9)	192 (12.0)	19 (17.6)	129 (10.7)	5 (10.0)
**missing**	461 (11.4)	42 (2.6)	1 (0.9)	30 (2.5)	2 (4.0)
**frequent medication intake**^d^**, N (%)**					
**yes**	74 (1.8)	105 (6.6)	54 (50.0)	33 (2.7)	15 (30.0)
**no**	3442 (85.4)	1337 (83.5)	41 (38.0)	1056 (87.8)	30 (60.0)
**missing**	514 (12.8)	159 (9.9)	13 (12.0)	114 (9.5)	5 (10.0)

^a ^Drinking (yes) was defined as daily or almost daily drinking of alcoholic beverage and drinking (no) was defined as no or casual drinking of alcoholic beverage.

^b ^Smoking (yes) was defined as current smoking and smoking (no) was defined as never or past smoking.

^c ^High education was defined as completion high school or University and low as any other response.

^d ^Frequent medication intake was defined as intake of acute pain drugs on ≥15 days per month.

On average respondents of both groups, CM and CTTH were older than those in the episodic headache groups. CM group comprised predominantly of females and in CTTH group the gender distribution was equal.

Tables 2, 3 and 4 show the results of ORs and 95%CIs of possible risk factors for CM vs. CTTH (Table 2) and CM vs. NH and CTTH vs. NH (Table 3) and finally CM vs. EM, and CTTH vs. ETTH (Table 4).

**Table 2:** Association between possible risk factors and CM vs. CTTH

		**Crude**		**Model 1**^a^		**Model 2**^b^	
		**OR**	**95%CI**	**OR**	**95%CI**	**OR**	**95%CI**
**CM vs. CTTH**	age	0.98	0.95-1.00	0.98	0.95-1.01	0.99	0.96-1.02
	female vs male	2.95	1.47-5.94	2.24	1.02-4.91	2.34	1.00-5.49
	smoking vs non-smoking	1.34	0.66-2.70	1.52	0.70-3.30	1.81	0.76-4.34
	drinking vs non-drinking	0.29	0.11-0.76	0.38	0.13-1.11	0.31	0.09-1.04
	obese vs not obese	1.86	0.65-5.31	2.50	0.78-7.98	1.85	0.54-6.27
	low vs high education	1.36	0.60-3.06	1.14	0.46-2.80	0.72	0.27-1.97
	frequent vs no frequent medication intake	2.63	1.26-5.53			1.68	0.73-3.88

^a ^model 1: fully adjusted without frequent medication intake.

^b ^model 2: fully adjusted with frequent medication intake.

**Table 3:** Association between possible risk factors and CM vs. NH / CTTH vs. NH

		**Crude**		**Model 1**^a^		**Model 2**^b^	
		**OR**	**95%CI**	**OR**	**95%CI**	**OR**	**95%CI**
**CM vs NH**	age	0.99	0.98-1.01	0.98	0.97-1.00	0.98	0.95-1.00
	female vs male	4.04	2.63-6.22	4.25	2.70-6.66	2.64	1.57-4.45
	smoking vs non-smoking	1.70	1.15-2.52	1.88	1.25-2.83	1.77	1.07-2.94
	drinking vs non-drinking	0.46	0.22-0.95	0.74	0.35-1.57	0.64	0.25-1.64
	obese vs not obese	1.39	0.84-2.31	1.72	1.02-2.92	0.85	0.43-1.70
	low vs high education	1.67	1.02-2.73	1.48	0.88-2.50	0.85	0.47-1.54
	frequent vs no frequent medication intake	61.26	38.41-97.70			59.75	34.74-102.77
**CTTH vs NH**	age	1.02	0.99-1.04	1.02	0.99-1.04	1.00	0.97-1.03
	female vs male	1.37	0.78-2.40	1.48	0.82-2.68	1.11	0.58-2.13
	smoking vs non-smoking	1.28	0.71-2.31	1.37	0.75-2.51	1.06	0.53-2.10
	drinking vs non-drinking	1.61	0.82-3.17	1.67	0.82-3.43	2.28	1.06-4.93
	obese vs not obese	0.75	0.30-1.90	0.71	0.27-1.81	0.42	0.15-1.18
	low vs high education	1.23	0.64-2.36	1.11	0.56-2.22	1.17	0.54-2.53
	frequent vs no frequent medication intake	23.26	12.01-45.05			30.03	14.38-62.71

^a ^model 1: fully adjusted without frequent medication intake.

^b ^model 2: fully adjusted with frequent medication intake.

**Table 4:** Association between possible risk factors and CM vs. EM / CTTH vs. ETTH

		**Crude**		**Model 1**^a^		**Model 2**^b^	
		**OR**	**95%CI**	**OR**	**95%CI**	**OR**	**95%CI**
**CM vs EM**	age	1.04	1.02-1.05	1.03	1.01-1.05	1.04	1.01-1.06
	female vs male	1.12	0.72-1.74	1.09	0.69-1.72	0.98	0.58-1.65
	smoking vs non-smoking	1.48	0.99-2.21	1.62	1.06-2.46	1.43	0.88-2.32
	drinking vs non-drinking	1.14	0.54-2.41	1.12	0.52-2.42	1.29	0.51-3.26
	obese vs not obese	1.54	0.92-2.58	1.40	0.82-2.38	0.99	0.52-1.89
	low vs high education	2.32	1.41-3.81	1.69	1.00-2.87	0.94	0.53-1.68
	frequent vs no frequent medication intake	16.77	10.67-26.35			15.17	9.29-24.77
**CTTH vs ETTH**	age	1.06	1.03-1.08	1.05	1.02-1.08	1.04	1.01-1.07
	female vs male	0.67	0.38-1.78	0.70	0.38-1.30	0.67	0.34-1.35
	smoking vs non-smoking	1.27	0.70-2.32	1.32	0.71-2.45	0.85	0.41-1.77
	drinking vs non-drinking	2.69	1.34-5.41	2.10	0.99-4.49	3.08	1.34-7.09
	obese vs not obese	0.94	0.37-2.42	0.71	0.27-1.87	0.37	0.12-1.16
	low vs high education	2.08	1.08-4.03	1.65	0.82-3.31	1.64	0.75-3.57
	frequent vs no frequent medication intake	16.00	7.87-32.55			20.47	8.84-47.36

^a ^model 1: fully adjusted without frequent medication intake.

^b ^model 2: fully adjusted with frequent medication intake.

Participants with CM compared to CTTH were more likely to be female (OR: 2.34, 95%CI: 1.00-5.49) and less likely to drink (OR: 0.31, 95%CI: 0.09-1.04). By trend participants with CM were more likely to smoke (OR: 1.81, 95%CI: 0.76-4.34), to be obese (OR: 1.85, 95%CI: 0.54-6.27), to report frequent intake of acute pain drugs (OR: 1.68, 95%CI: 0.73-3.88) and less likely to be low educated (OR: 0.72, 95%CI: 0.27-1.97) compared to CTTH (Table 2).

Participants with CM compared to NH were more likely to be female (OR: 2.64, 95%CI: 1.57-4.45), to smoke (OR: 1.77, 95%CI: 1.07-2.94) and to report frequent intake of acute pain drugs (OR: 59.75, 95%CI: 34.74-102.77) and by trend they are less likely to drink (OR: 0.64, 95%CI: 0.25-1.64). Participants with CTTH compared to NH were more likely to drink (OR: 2.28, 95%CI: 1.06-4.93) and to report frequent intake of acute pain drugs (OR: 30.03, 95%CI: 14.38-62.71) and by trend they were more likely to be female (OR: 1.11, 95%CI: 0.58-2.13), to be low educated (OR: 1.17, 95%CI: 0.54-2.53) and less likely to be obese (OR: 0.42, 95%CI: 0.15-1.18) (Table 3).

Participants with CM compared to EM were more likely to be older (OR: 1.04, 95%CI: 1.01-1.06) and to report frequent intake of acute pain drugs (OR: 15.17, 95%CI: 9.29-24.77) and by trend they were more likely to smoke (OR: 1.43, 95%CI: 0.88-2.32) and to drink (OR: 1.29, 95%CI: 0.51-3.26). Participant with CTTH compared to ETTH were more likely to be older (OR: 1.04, 95%CI: 1.01-1.07), to drink (OR: 3.08, 95%CI: 1.34-7.09) and to report frequent intake of acute pain drugs (OR: 20.47, 95%CI: 8.84-47.36) and by trend they were more likely to be male (OR: 0.67, 95%CI: 0.34-1.35) and to be low educated (OR: 1.64, 95%CI: 0.75-3.57) (Table 4).

Adjusting for frequent intake of acute pain drugs significantly changed the models, whereby frequent intake of acute pain drugs became the strongest risk factor.

## Discussion

In this study we aimed to identify specific risk factors for the development of chronic migraine and chronic tension-type headache in order to support the clinical distinction between these two most common primary headache disorders on the basis of their respective epidemiological risk factor profile. The prevalence of migraine in our study was 17.7% (1.1% CM + 16.6% EM). This is comparable to previous studies from Europe and the USA [6-10] which reported a prevalence of 13.2% to 21.3%. Previous data on the prevalence of TTH are more heterogeneous and differ from 20.7% to more than 63% [10-13]. In our study the prevalence of TTH was lower with 13% (0.5% CTTH + 12.5% ETTH). The wide variation of estimated prevalence in TTH might be due to different questionnaires used, the improper distinction between life-time and one-year prevalence and different age profiles.

Overall the one-year prevalence of chronic headache in our study was 2.6% (95%CI: 2.3-3). The results were in line with European and American studies which reported a prevalence of 2.98% to 4.7% [14-19]. In our study CM was more common and comprised of more women than CTTH. Compared to other European and American studies the prevalence of CM with 1.1% (95%CI: 0.9-1.3) and CTTH with 0.5% (95%CI: 0.4-0.7) in our study was lower and more men were affected. For CM the prevalence reported by other European and American studies was 1.4% to 2.4% and 2% to 2.2% for CTTH [14,15,18,20]. The reported fraction of women was 89% in CM and 91% in CTTH [14]. Noticeable is, that Scher et al. found CTTH more often than CM [18]. The higher prevalence of CM and CTTH in the literature might be due to the fact, that different definitions for headache subtypes were used.

Our study demonstrated different risk factors comparing CM and CTTH which may reflect a different neurobiology of both conditions. Participants with CM compared to CTTH were more likely to be female and less likely to drink alcohol. By trend participants with CM are more likely to smoke, to be obese, to report frequent intake of acute pain drugs to treat acute headache attacks and less likely to be low educated compared to CTTH.

In our study participants with CM drank alcohol less frequently than individuals without headache or other investigated headache subgroups. The same was reported in the literature [21]. It was also reported that consumption of alcoholic beverages aggravates or triggers migraine, which is a good explanation for this observation [22-24]. The less alcohol preference of CM participants is attributed to the role of migraine trigger that alcoholic beverages can exert, but it does not account for the higher use of alcoholic drinks among CM participants compared to EM participants in our study population (OR: 1.29, 95%CI: 0.51-3.26) (Table 4). In theory, CM patients should avoid alcoholic drinks more than EM patients. Participants with CTTH were more likely to drink than participants with CM or NH (CM vs. CTTH: OR: 0.31, 95%CI: 0.09-1.04, CTTH vs. NH: OR: 2.28, 95%CI: 1.06-4.93). Daily or almost daily drinking of alcoholic beverages might be a useful feature in clinical distinction of CM and CTTH.

A higher severity of headaches among smokers than among non-smokers was reported previously, but current data are still insufficient to prove a causal link between cigarette smoking and worsening headache [25,26]. Otherwise, headache is a known withdrawal syndrome of nicotine cessation [27]. In our study participants with CM were more likely to smoke than participants with CTTH (OR: 1.81, 95%CI: 0.76-4.34). Participants with CM were also more likely to smoke than participants with EM (OR: 1.43, 95%CI: 0.88-2.32) or NH (OR: 1.77, 95%CI: 1.07-2.94). Participants with CTTH were not more likely to smoke than ETTH (OR: 0.85, 95%CI: 0.41-1.77) or NH (OR: 1.06, 95%CI: 0.53-2.10) in our adjusted model (model 2).

Obesity was found to be a risk factor for CM in the literature, although migraine by itself was not more prevalent in obese patients. Obesity was also associated with the frequency and severity of migraine [28], but the causal link between obesity and migraine remains to be determined. There are studies that reported an association between obesity and CM that did not adjust for frequent intake of acute pain drugs [29,30]. After adjusting for frequent intake of acute pain drugs the association between CM and obesity compared to NH diminished (OR: 0.85; 95%CI: 0.43-1.70). Adjustment for frequent intake of acute pain drugs changed the model significantly, and resulted in frequent intake of acute pain drugs becoming the strongest risk factor for the development of CM. However, our data suggests a trend that participants with CM compared to CTTH were more likely to be obese (OR: 1.85, 95%CI: 0.54-6.27). This may hint at a common pathophysiological influence of obesity and the development of chronic headache independent of the underlying primary headache disorder. This association or influence might be pronounced differently in different headache conditions and therefore appears to be stronger in CM compared to CTTH.

An association between low education and CH is frequently discussed in the literature. Studies showed that the prevalence of CH was highest in the lowest education category [18] and subjects with CTTH had less education, on average, than subjects with ETTH [20]. This is consistent with our results in regard to CTTH and education. Participants with CTTH are lower educated than participants with NH (OR: 1.17, 95%CI: 0.54-2.53), ETTH (OR: 1.64, 95%CI: 0.75-3.57) and also CM (CM vs. CTTH: OR: 0.72, 95%CI: 0.27-1.97). Regarding CM reports in the literature are inconsistent [18,31] but generally describe an association between CM and low education after fully adjusting. Crude and in model 1 we found that participants with CM were more likely to be low educated compared to NH or EM. However, adjusting for frequent intake of acute pain drugs (model 2) changes the model significantly and we found no association between low education and CM (CM vs. NH: OR: 0.85, 95%CI: 0.47-1.54; CM vs. EM: OR: 0.94, 95%CI: 0.53-1.68) any more, whereas frequent intake of acute pain drugs became the strongest risk factor. Many studies which describe an association between CM and low education did not adjust for frequent intake of acute pain drugs and might have been misinterpreted [29,31]. The role of education for the development of chronic daily headache needs to be further elucidated in future research.

A strong association between CH and overuse of acute pain drugs was demonstrated in many epidemiologic studies worldwide [14,15,32-35]. Overuse of acute pain drugs was found in more than half of CH patients in clinic-based studies. This was confirmed by our data with 50% of CM and 30% of CTTH reporting frequent intake of acute pain drugs. It is unknown if frequent intake of acute pain drugs is the cause or consequence of CH. Compared to CTTH there is a trend that participants with CM were more likely to report frequent intake of acute pain drugsn (OR: 1.68, 95%CI: 0.73-3.88).

In summary, we found lower prevalence of CDH, CM and CTTH and a higher male fraction in our study population compared to the literature. Furthermore, we cannot confirm an association between obesity and CM, as well as low education and CM in the fully adjusted model. Different smoking, drinking and use of acute pain drugs habits as well as differences in weight and gender between CM and CTTH were found.

Strengths of this study are the large representative population-based sample in three different regions of Germany and therefore the large number of outcomes. The power was great enough to test a number of predefined covariates. Another strength is the use of validated questionnaires that were published prior to the study.

Limitations of our study are the overall response rate of the survey of 55%, which is satisfactory in comparison to other large-scale population-based studies in Western Europe and the US, but selection bias cannot be excluded. More affected persons tend to participate more eagerly than non-affected healthy persons and very old and very young people often do not participate in surveys. Another limitation is the retrospective design of detecting the headache features, recall bias cannot be excluded. These limitations are not unique to our study. All large-scale questionnaire-based studies face these challenges.

## Conclusion

In conclusion, our investigation shows that CM and CTTH have slightly different risk factors that might be due to their different neurobiology and may help in the clinical distinction of CH into CM and CTTH. Patients with CM were more likely to be female, to smoke, to be obese, and to report frequent intake of acute pain drugs, while patients with CTTH had a higher intake of alcoholic beverages and a lower education. Consideration of these different epidemiological profiles may become an asset in finding the correct primary headache disorder in patients presenting with chronic headache and guide appropriate treatment.

## Competing interests

Min-Suk Yoon, Sara Schramm and Susanne Moebus have nothing to disclose. Mark Obermann has received scientific support and/or honoraria from Biogen Idec, Novartis, Sanofi-Aventis, Pfizer, and Teva. He received research grants from the German ministry for education and research (BMBF). Hans-Christoph Diener has been a consultant for and is a member of the speakers’ bureau of Allergan, Inc. Zaza Katsarava has received grants and research support, been a consultant for, and is a member of the speakers’ bureau of Allergan, Inc.

## Authors’ contributions

SS performed the statistical analysis and draft the manuscript. MO participated in the design of the study and helped to draft the manuscript. ZK conceived of the study, and participated in its design and coordination and helped to draft the manuscript. HD conceived of the study, and participated in its design and helped to draft the manuscript. SM participated in the design of the study and helped to draft the statistical analysis. MY participated in the design of the study and coordination and helped to draft the manuscript. All authors read and approved the final manuscript.
